# Stradivari harp tree-ring data

**DOI:** 10.1016/j.dib.2022.108453

**Published:** 2022-07-11

**Authors:** Mauro Bernabei, Jarno Bontadi, Luigi Sisto

**Affiliations:** a CNR-IBE, Institute for BioEconomy, National Research Council, via Biasi 75, 38098 San Michele all'Adige, Trento, Italy; b Museo Storico Musicale, Conservatorio di Musica San Pietro a Majella, Via San Pietro a Majella 35, 80138 Naples, Italy

**Keywords:** Stradivari, Amati, Harp, Cello, Dendrochronology, Violin-maker, Musical instruments, Resonant wood

## Abstract

Three tree ring sequences were collected on the soundboard of the Stradivari harp. Due to the presence of the strings in the centre of the harp soundboard, the sampling of the tree ring widths was focused separately on the right side (RX), the central (CX) and the left side (LX). Tree ring measurements were carried out by using the Video Time Table (VTT), an instrument that combines a portable measuring device and a digital, high-resolution video camera. The VTT allowed non-invasive measurements of the tree rings to be made in situ and to immediately verify the quality of the sampling. The growth rings of the central portion were sampled using a high-resolution camera, which made it possible to bypass the barrier formed by the neck and strings. The consequent parallax and focus problems were overcome by taking many photographs from different angles. The measurements on the photographs were made with the CooRecorder program. The dendrochronological data were acquired with the PAST4 program and graphically processed and analysed with the PAST4 and 5 programs.

## Specifications Table

**Table:** 

Subject	Art and Humanities
Specific subject area	The subject area is that of the history of music and violin making. The data presented here may be useful for dating other musical instruments by Stradivari or other important violin makers from the golden age of Italian violin making in the 17^th^ and 18^th^ centuries.
Type of data	The data presented here are numerical and represent the tree ring width in mm/100. They are in Heidelberg format (*.fh).The Format Heidelberg is a text based file format that was introduced by Frank Rinn with his TSAP software. Format Heidelberg files may contain one or more records. Each record consists of a header and ring width data.The .fh files can be used by the most common dendrochronology programmes or can be transformed into any other format thanks to the TRiCYCLE program http://www.tridas.org/tricycle/
How the data were acquired	Part of the data (RX and LX) were acquired via Video Time Table (VTT), an instrument that combines a portable measuring device and a digital, high-resolution video camera. CX were acquired via high-resolution digital photographs.The Video Time Table [1] is an instrument that combines a portable measuring device and a digital, high-resolution video camera.The device has the following advantages: - The tree rings can be measured on site; - The measurements are not invasive; - The correctness of the measurements can be checked immediately.The measuring device consists of four fundamental parts: a tripod, the optics, a three-axes movement device and an external unit for the movement's control. The whole system is connected to a portable computer that analyses the data. The optics consist of a digital video camera with a focal distance of 20 cm, which avoids any direct contact with the wooden object. The three-axes movement device allows movements with a precision of 1/8000 mm. The tree-ring series obtained can be visualized and processed with the PAST4 software of SCIEM (Scientific Engineering and Manufacture). The VTT's video control enables its user to save the most important images, a service that has proved to be very useful in those cases where doubts arose regarding the interpretation of a sequence.The measurements on the photographs were made with the CooRecorder program (Cybis, Sweden) [2].
Data format	Raw
Description of data collection	Three tree-ring series were obtained from the soundboard of the Stradivari harp: one on the left (LX), one in the centre (CX) and one on the right side (RX). They were repeated at various parts of the belly of the instrument in order to maximize the number of growth rings available and, at the same time, to avoid errors caused by possible distortions in the veining. The possibility of immediate comparisons between the dendrochronological series measured allowed the repetition of a measurement whenever anomalies were detected in the tendency of a ring curve. The digital photographs permitted a constant comparison between the wooden surface analysed and the dendrochronological series recorded.The species is spruce (*Picea abies* Karst.).
Data source location	Institution: Museum of the Conservatorio San Pietro a Majella,City/Town/Region: NaplesCountry: ItalyLatitude and longitude (and GPS coordinates, if possible) for collected samples/data:Latitude: 40.849241, Longitude: 14.25263
Data accessibility	Host. Mendeley DataRepository name: Stradivari harp tree ring dataData identification number: V2Direct URL to data:https://data.mendeley.com/datasets/hwfg44dfbyDOI:10.17632/hwfg44dfby.2
Related research article	M. Bernabei, J. Bontadi, L., Sisto, Dendrochronological analysis of the Stradivari's harp, Dendrochronologia. 74 (2022) 125960. https://doi.org/10.1016/j.dendro.2022.125960

## Value of the Data

• The data provide a valuable reference chronology, useful to dendrochronologists for dating musical instruments and other material of cultural-historical interest;
• the harp tree-ring series can be used for comparison with those of other instruments by Stradivari or other violin-makers from the golden age of classical Italian violin-making (16^th^-18^th^ centuries);
• data on the harp tree rings can help identify the region from which Antonio Stradivari and Nicola Amati sourced the wood for their instruments;
• historians, musicologists, dendrochronologists, violin makers will be able to benefit from these original data, dated to the calendar year, to analyse and attribute other musical instruments.
• A further possible field of application concerns climatic or environmental reconstructions, thanks to the capacity of tree rings to report information on past climate with annual precision.

## 1 Data Description

Three tree-ring series were obtained from the soundboard of the Stradivari harp [3,4]: one on the left (LX), one on the centre (CX) and one on the right side (RX). The data represent the tree ring width in mm/100. They are in Heidelberg format (*.fh).

The Format Heidelberg is a text based file format that was introduced by Frank Rinn with his TSAP software. Format Heidelberg files may contain one or more records. Each record consists of a header and ring width data. The .fh files can be used by the most common dendrochronology programmes or can be transformed into any other format thanks to the TRiCYCLE program http://www.tridas.org/tricycle/

## 2 Experimental Design, Materials and Methods

The Stradivari's harp [5] is the only surviving portable diatonic harp [4], now in the possession of the Museum of the Conservatorio San Pietro a Majella, Naples. The harp bears the inscription “*Ant:° Stradivarivs Cremonen.s F. 1681*” engraved on the column [6]. This small harp is a “*unicum*” among the approximately 550 surviving instruments of the Cremonese craftsman. Its dimensions are 45 cm for the neck, 75 cm is the soundbox length, and 93 cm for the column [7].

Part of the data (RX and LX) were acquired on the soundboard of the harp via Video Time Table (VTT), an instrument that combines a portable measuring device and a digital, high-resolution video camera [8]. The central sequence (CX) was acquired via high-resolution digital photographs.

**Fig. 1:**
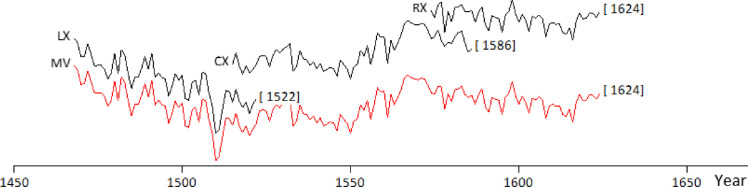
The three tree ring series collected on the Stradivari harp and their mean value (in red). The tree ring sequences are stacked one above the other on a single axis (year) to facilitate the visual comparison using a logarithmic scale. (For interpretation of the references to color in this figure legend, the reader is referred to the web version of this article.)

The Video Time Table (VIAS, 2005) has the following advantages: - The tree rings can be measured on site; - The measurements are not invasive; - The correctness of the measurements can be checked immediately. The measuring device consists of four fundamental parts: a tripod, the optics, a three-axes movement device and an external unit for the movement's control. The whole system is connected to a portable computer that analyses the data. The optics consist of a digital video camera with a focal distance of 20 cm, which avoids any direct contact with the wooden object. The three-axes movement device allows movements with a precision of 1/8000 mm. The tree-ring series obtained can be visualized and processed with the PAST4 software of SCIEM [9]. The VTT's video control enables its user to save the most important images, a service that has proved to be very useful in those cases where doubts arose regarding the interpretation of a sequence. The measurements on the photographs were made with the CooRecorder program [2]. The consequent parallax and focus problems were overcome by taking many photographs from different angles. The final averaged tree ring sequence, representative of the Stradivari's harp, spans 157 rings/years and is dated 1468-1624 (Fig. 1).

## Ethics Statements

Our work did not involve human subjects;

Our work did not involve animal experiments;

Our work did not involve data collected from social media platforms;

## CRediT Author Statement

CRediT is an initiative that enables authors to share an accurate and detailed description of their diverse contributions to a published work.

Example of a CRediT author statement:

**Bernabei Mauro:** Conceptualization, Methodology, Data curation, Writing – original draft, Writing – review and editing; **Bontadi Jarno**: Conceptualization, Data curation, Formal analysis, Methodology; **Sisto Luigi**: Conceptualization, Project administration, Supervision.

## Declaration of Competing Interest

The authors declare that they have no known competing financial interests or personal relationships that could have appeared to influence the work reported in this paper.

## Data Availability

Stradivari harp (Original data) (Mendeley Data).

## Appendix Supplementary materials

Image, application 1
